# Fixed-Time Synchronization of Coupled Oscillator Networks with a Pacemaker

**DOI:** 10.3390/s22239460

**Published:** 2022-12-03

**Authors:** Xiufeng Guo, Pengchun Rao, Zhaoyan Wu

**Affiliations:** 1 School of Science, East China Jiaotong University, Nanchang 330013, China; 2 School of Mathematics and Statistic, Jiangxi Normal University, Nanchang 330022, China

**Keywords:** fixed-time, Kuramoto oscillator, pacemaker, cyber-physical system (CPS), synchronization

## Abstract

This paper investigates the fixed-time synchronization problem of a Kuramoto–oscillator network in the presence of a pacemaker. Based on the framework of the cyber-physical system (CPS), fixed-time synchronization criteria of such network are presented respectively for identical and non-identical oscillators. In virtue of Lyapunov stability analyses, sufficient conditions are deduced for achieving phase agreement and frequency synchronization for arbitrary initial phases and/or frequencies under distributed control strategies. Theoretical analysis shows that synchronization can be achieved in a fixed time, which is unrelated to initial phases/frequencies. Furthermore, the upper bounds of synchronization time are also obtained. Finally, the numerical simulations also verify the effectiveness of the derived results.

## 1. Introduction

In recent years, synchronous emergence of complex dynamical networks [1,2,3,4,5,6,7,8], as a representative class of self-organized synergistic behavior, has aroused numerous attention from many researchers in various fields, such as biology [1,2], engineering [3,4,5,6], physics [7,8], and so on. People have witnessed such phenomenon in biology and physics including flashing fireflies [1], chorus of crickets [2], Josephson junctions [7], and the rhythmic applause in the theater [8]. In the past several decades, the Kuramoto model [9,10] has become a hot topic in the control community. By now, many researchers [11,12] have devoted to derive synchronization criteria subject to coupling strength and initial phases for Kuramoto–oscillator networks. In [11], Jadbabaie considered a connected, undirected, and identical Kuramoto–oscillator network and presented that the phase agreement is achieved when the initial phases lie within the interval $\left(-\frac{\pi }{2},\frac{\pi }{2}\right)$. Chopra and Song [12] pointed out that frequency synchronization is achieved if the initial phase differences of each oscillator are within the interval $\left(-\frac{\pi }{2},\frac{\pi }{2}\right)$.

Considering the impact of the pacemaker (the so-called leader) on Kuramoto oscillators is of significance from both theoretical and practical points of views. For example, in the clock synchronization of wireless networks [13], the time references in individual node are synchronized through cell-to-cell interactions and external coordination from a time base. Therefore, Kuramoto oscillators with a pacemaker have attracted considerable attention. In [14,15,16,17], researchers derived fruitful results by taking the pacemaker into consideration. In addition, based on the framework of cyber-physical system [18,19], the researchers in [20,21] used distributed linear controllers to synchronize the Kuramoto–oscillator network. The derived stability conditions were independent on the initial phases, which indicates that global synchronization was achieved. In [21], on the basis of such distributed linear control strategy, a sufficient criterion for the Kuramoto–oscillator network with a pacemaker was established. However, the above-mentioned synchronziation belongs to asymptotically synchronization, which means that synchronization is achieved when time approaches infinity.

Additionally, many researchers have focused on synchronization time of complex networks [22,23,24,25,26,27,28,29]. In [22], Shi and Ma presented a unified theoretical method to obtain the finite/fixed-time synchronization for complex networks with uncertain inner coupling by adopting the quantized controller and the quantized adaptive controller. Aouiti and Assali [23] obtained novel and effective criteria for achieving the finite-time synchronization. In [24], sufficient conditions were obtained to synchronize the nodes in each cluster to desired states in a settling time. Several fixed-time synchronization criteria expressed by some linear matrix inequalities are presented [25]. Zheng et al. [26] investigated the fixed-time synchronization of discontinuous competitive neural networks with time-varying delays and made the upper bound of the settling time less conservativeness. In [27], Wu and Li studied the finite-time and fixed-time synchronization of Kuramoto–oscillator network by adopting a novel multiplex controller. Based on a cyber-physical system, the finite-time synchronization of a Kuramoto–oscillator network with a pacemaker was studied [29]. Compared with the finite-time synchronization, the settling time in fixed-time synchronization can be adjusted to the desired value independent on initial states, which is of great practical importance. However, up to now, there are few results on fixed-time synchronization of a Kuramoto–oscillator network in the presence of a pacemaker.

Motivated by the above discussions, it is significant to consider the fixed-time synchronization of Kuramoto–oscillator network with a pacemaker. In this paper, based on CPS, we explore fixed-time synchronization criteria of such network by adopting distributed control strategies.

Our contributions are summarized as follows:

1. Effective criteria are established to deal with fixed-time phase agreement and frequency synchronization for the Kuramoto–oscillator network with a pacemaker, and upper bounds of synchronization time are also provided;
2. Compared with our previous results [29], synchronization can be achieved for arbitrary initial phases/ frequencies and the upper bounds of synchronization time are not affected by initial phases/ frequencies;
3. The requirement on the connectivity of physical system is relaxed.

The remainder of this paper is displayed as follows: Section 2 presents the detailed model description as well as preliminaries. In Section 3, sufficient conditions for fixed-time phase agreement and fixed-time frequency synchronization are derived for identical and non-identical oscillators, respectively. Section 4 provides numerical examples to verify the theoretical results, and Section 5 concludes the whole paper.

## 2. Model and Preliminaries

A Kuramoto–oscillator network consisting of *N* oscillators with control input $u_{i}$ is described as

(1) $${\dot{\theta }}_{i}=\omega_{i}+\sum_{j=1}^{N}a_{ij}\sin\left(\theta_{j}-\theta_{i}\right)+u_{i},\;i\in I,$$

where $I=\left\{1,\cdots ,N\right\}$, $\theta_{i}$ and $\omega_{i}$ are the phase and natural frequency of oscillator *i*, respectively. $A=\left[a_{ij}\right]\in R^{N\times N}$ denotes the adjacency matrix of an undirected network, where $a_{ij}=a_{ji}=1\;\left(i\neq j\right)$ if and only if there is an edge between oscillators *i* and *j*; otherwise, $a_{ij}=0$. Let $L_{A}=D^{A}-A$ be the Laplacian matrix associated with the adjacency matrix *A*, where $D^{A}\in R^{N\times N}$ is a diagonal matrix with $D_{ii}^{A}=\sum_{j=1}^{N}a_{ij}\;\left(\forall i\in I\right)$. The network associated with the adjacency matrix *A* is called the physical network.

Assume that there is a pacemaker with dynamics

$${\dot{\theta }}_{0}=\omega_{0},$$

where $\theta_{0}$ and $\omega_{0}$ are the phase and natural frequency of the pacemaker, respectively.

In this paper, we focus on phase agreement and frequency synchronization with respect to the pacemaker in a fixed time.

**Definition** **1.** *Network (1) with control input $u_{i}$ achieves (pacemaker-based) fixed-time phase agreement, if there exists a settling time T>0 independent on the initial states $\theta_{i}\left(0\right)\;\left(i\in \left\{0\right\}⋃I\right)$, such that*

(2) $$\begin{array}{c} \lim_{t\to T}\left(\theta_{i}-\theta_{0}\right)=0,\;i\in I, \end{array}$$

*and $\theta_{i}-\theta_{0}\equiv 0$ for t≥T.*

**Definition** **2.** *Network (1) with control input $u_{i}$ achieves (pacemaker-based) fixed-time frequency synchronization, if there exists a settling time T>0 independent on the initial states ${\dot{\theta }}_{i}\left(0\right)\;\left(i\in \left\{0\right\}⋃I\right)$, such that*

(3) $$\lim_{t\to T}\left({\dot{\theta }}_{i}-{\dot{\theta }}_{0}\right)=0,\;i\in I,$$

*and ${\dot{\theta }}_{i}-{\dot{\theta }}_{0}\equiv 0$ for t≥T.*

In order to obtain sufficient conditions for the above-mentioned synchronization, the following Lemmas are needed.

**Lemma** **1.** *[30] Let $A=\left[a_{ij}\right]\in R^{N\times N}$ and $L_{A}$ be the adjacency matrix and Laplacian matrix of an undirected graph G with N nodes, respectively. $x^{T}L_{A}x=\frac{1}{2}\sum_{i=1}^{N}\sum_{j=1}^{N}a_{ij}{\left(x_{i}-x_{j}\right)}^{2}$ holds, where $x={\left(x_{1},x_{2},\cdots ,x_{N}\right)}^{T}$.*

**Lemma** **2.** *[31] Consider the dynamic system defined by the following differential equation:*

(4) $$\dot{x}\left(t\right)=f\left(t,x\left(t\right)\right),\;x\left(0\right)=x_{0},\;t\in R_{+},$$

*where $x\left(t\right)\in R^{n\times 1}$ is the state vector and $f:R_{+}\times R^{n\times 1}\to R^{n\times 1}$ is a nonlinear function.*

*If there exists a continuous radially unbounded function V: $R^{n\times 1}\to R_{+}\cup \left\{0\right\}$ such that*

*(1) V(x(t))=0⇔x(t)=0;*

*(2) Any solution x(t) of (4) satisfies the inequality*

$$D^{+}V\left(x\left(t\right)\right)\leq -{\left(\alpha V^{p}\left(x\left(t\right)\right)+\beta V^{q}\left(x\left(t\right)\right)\right)}^{k},$$

*for some parameters α, β, p, q, k>0 with pk<1 and qk>1, where $D^{+}V\left(x\left(t\right)\right)$ denotes the upper right-hand derivative of the function V(x(t)). Then, the origin is globally fixed time stable for system (4) and the settling time bounded by*

$$T\leq \frac{1}{\alpha^{k}\left(1-pk\right)}+\frac{1}{\beta^{k}\left(qk-1\right)}.$$

For a real symmetric matrix L, let $\lambda_{\min}\left(L\right)$ be the minimum eigenvalue of matrix L. Denote $sig{\left(x\right)}^{\alpha }=sign\left(x\right){\left|x\right|}^{\alpha }$, where the signum function sign(x) is defined as

$$\mathrm{sign}\left(x\right)=\left\{\begin{array}{c} \;1,\;x>0,\\ \;0,\;x=0,\\ -1,\;x<0. \end{array}\right.$$

In addition, denote the phase differences with respect to the pacemaker as

$$\xi_{i}=\theta_{i}-\theta_{0}.$$

## 3. Fixed-Time Phase Agreement and Frequency Synchronization

In this section, based on CPS, we move to seek sufficient criteria for fixed-time phase agreement and fixed-time frequency synchronization by designing distributed controllers.

### 3.1. Fixed-Time Phase Agreement

In this subsection, we consider the case of all oscillators with identical natural frequency, i.e., $\omega_{i}=\omega_{0},\;\forall i\in I$. Thus, network (1) becomes

(5) $${\dot{\theta }}_{i}=\omega_{0}+\sum_{j=1}^{N}a_{ij}\sin\left(\theta_{j}-\theta_{i}\right)+u_{i},\;i\in I.$$

For achieving fixed-time phase agreement, based on CPS, we design a distributed control strategy $u_{i}$ as follows:

(6) $$u_{i}=\sum_{j=1}^{N}b_{ij}\left(\theta_{j}-\theta_{i}\right)+f_{i}sig{\left(\theta_{0}-\theta_{i}\right)}^{\alpha_{1}}+g_{i}sig{\left(\theta_{0}-\theta_{i}\right)}^{\alpha_{2}},\;i\in I,$$

where $f_{i}>0$, $g_{i}>0$, $0<\alpha_{1}<1$, and $\alpha_{2}>1$. $B=\left[b_{ij}\right]\in R^{N\times N}$ denotes the adjacency matrix of an undirected network, where $b_{ij}=b_{ji}=1\;\left(i\neq j\right)$ if and only if there exists information exchange between oscillators *i* and *j*; otherwise, $b_{ij}=0$. The network associated with the connections between the oscillators in the controller (6) is called a cyber network. Let $L_{B}$ be the Laplacian matrix associated with the adjacency matrix *B*, where its elements are defined similarly to those of $L_{A}$.

**Theorem** **1.** *Network (5) under the distributed control strategy (6) achieves fixed-time phase agreement with the settling time bounded by*

(7) $$T_{1}\leq T_{0}\overset{\Delta }{=}\frac{1}{2^{\frac{\alpha_{1}-1}{2}}\left(1-\alpha_{1}\right)f_{\min}}+\frac{1}{2^{\frac{\alpha_{2}-1}{2}}\left(\alpha_{2}-1\right)g_{\min}},$$

*if*

$$\begin{array}{c} \lambda_{\min}\left(\cos\gamma \cdot L_{A}+L_{B}\right)\geq 0, \end{array}$$

*where γ∈(π,2π) satisies tanγ=γ, $f_{\min}=\min\left\{f_{1},\cdots ,f_{N}\right\}$, and $g_{\min}=\min\left\{g_{1},\cdots ,g_{N}\right\}$.*

**Proof** **of** **Theorem** **1.** See Appendix A. □

According to (7), we find that the upper bound of synchronization time is independent on initial states. By Theorem 1, it is sufficient to achieve fixed-time phase agreement if $\lambda_{\min}\left(\cos\gamma \cdot L_{A}+L_{B}\right)\geq 0$. Therefore, even if the physical network is not connected, phase agreement could be also achieved with the help of the cyber network, which relaxes the requirement on the connectivity of the physical network.

### 3.2. Fixed-Time Frequency Synchronization

Now, we further consider the case of all oscillators with non-identical natural frequencies, i.e., there exists some i∈I such that $\omega_{i}\neq \omega_{0}$.

For achieving fixed-time frequency synchronization, based on CPS, we design a distributed control strategy $u_{i}$ as follows:

(8) $$u_{i}=\sum_{j=1}^{N}b_{ij}\left(\theta_{j}-\theta_{i}\right)+U_{i},\;i\in I,$$

where ${\dot{U}}_{i}=f_{i}sig{\left({\dot{\theta }}_{0}-{\dot{\theta }}_{i}\right)}^{\alpha_{1}}+g_{i}sig{\left({\dot{\theta }}_{0}-{\dot{\theta }}_{i}\right)}^{\alpha_{2}}$, $f_{i}>0$, $g_{i}>0$, $0<\alpha_{1}<1$, $\alpha_{2}>1$, and $b_{ij}$ denotes the same as that in (6).

**Theorem** **2.** *Network (1) with non-identical oscillators under the distributed control strategy (8) achieves fixed-time frequency synchronization with the settling time bounded by*

(9) $$T_{2}\leq T_{0}\overset{\Delta }{=}\frac{1}{2^{\frac{\alpha_{1}-1}{2}}\left(1-\alpha_{1}\right)f_{\min}}+\frac{1}{2^{\frac{\alpha_{2}-1}{2}}\left(\alpha_{2}-1\right)g_{\min}},$$

*if*

$$\lambda_{\min}\left(L_{B}-L_{A}\right)\geq 0,$$

*where $L_{A}$ and $L_{B}$ represent the same matrices as those in Section 3.1.*

**Proof** **of** **Theorem** **2.** See Appendix B. □

According to (9), we find that the upper bound of synchronization time is independent on initial states. By Theorem 2, it is sufficient to achieve fixed-time frequency synchronization if $\lambda_{\min}\left(L_{B}-L_{A}\right)\geq 0$. Therefore, similar to Theorem 1, we also relax the requirement on the connectivity of the physical network.

**Figure 6:**
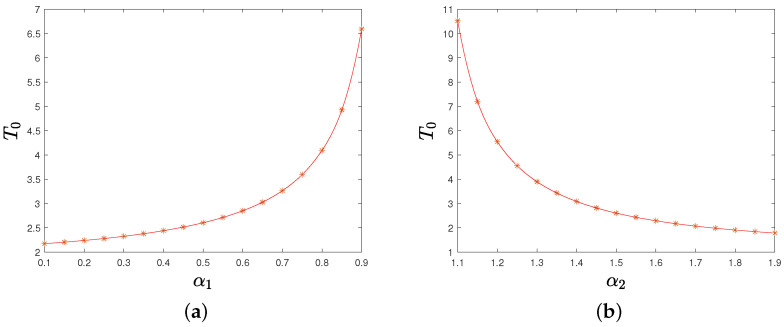
(**a**) The upper bounds of synchronization time $T_{0}$ under the distributed control strategy (6) for different parameters $\alpha_{1}$; (**b**) the upper bounds of synchronization time $T_{0}$ under the distributed control strategy (8) for different parameters $\alpha_{2}$.

**Remark** **1.** *By equations (7) and (9), the upper bounds of synchronization time $T_{0}$ increase as parameter $\alpha_{1}$ grows and decrease as parameter $\alpha_{2}$ grows both for identical and non-identical oscillators in the light of the monotonicity of $T_{0}$, which is also reflected in Figure 6 of Section 4. Additionally, $T_{0}$ decreases as $f_{\min}$ or $g_{\min}$ grows.*

**Remark** **2.** *In this paper, it is assumed that both physical and cyber networks are undirected. However, for directed networks, it is difficult to guarantee that the Laplacian matrices associated with physical and cyber networks are semi-definite ones. Consequently, it is a challenging work to extend the presented theoretical results to directed networks, which is one of our future directions.*

## 4. Numerical Simulation

**Figure 1:**
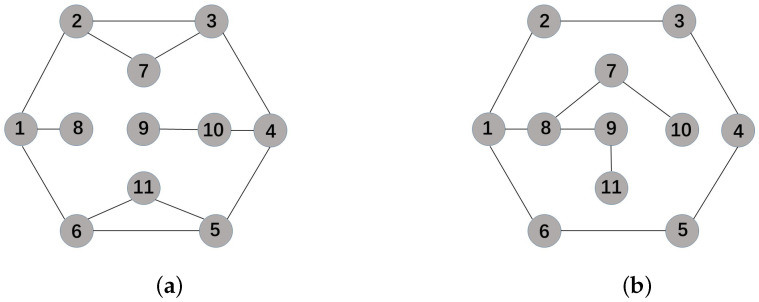
(**a**) Network associated with the adjacency matrix A; (**b**) network associated with the adjacency matrix B.

In this section, we assume networks associated with adjacency matrices *A* and *B* as shown in Figure 1a,b, respectively. In addition, $I=\left\{1,2,\cdots ,11\right\}$.

**Figure 2:**
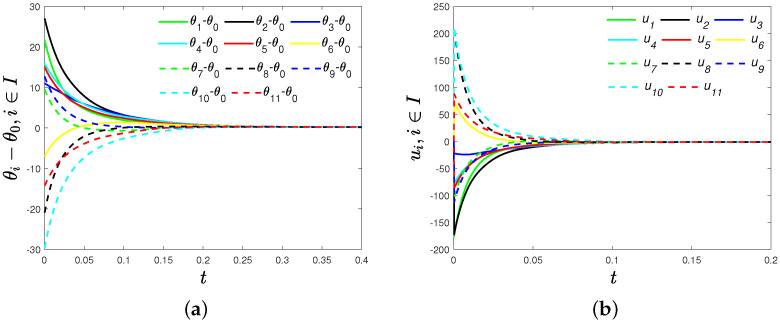
(**a**) Time evolutions of phase differences $\theta_{i}$-$\theta_{0}\;\left(i\in I\right)$ under the distributed control strategy (6); (**b**) time evolutions of the distributed control strategy (6).

We first verify Theorem 1. Set $\omega_{i}$= 1 (i∈{0}⋃I). Choose $\alpha_{1}=0.5$, $\alpha_{2}=1.5$ and $f_{i}=g_{i}=2$ (i∈I). Initial phases $\theta_{i}\left(0\right)$ (i∈{0}⋃I) are randomly chosen from the continuous distribution over [−10π,10π]. Obviously, $\lambda_{\min}\left(\cos\gamma \cdot L_{A}+L_{B}\right)=0$. By (7), we can calculate the upper bound of settling time $T_{0}$ = 2.8710 s. In Figure 2a, we can see phase difference $\theta_{i}-\theta_{0}$ (i∈I) converge to zero, and phase agreement is achieved about 0.2840 s < $T_{0}$, which means fixed-time phase agreement is achieved. Time evolutions of the distributed control strategy (6) of each oscillator are shown in Figure 2b.

**Figure 3:**
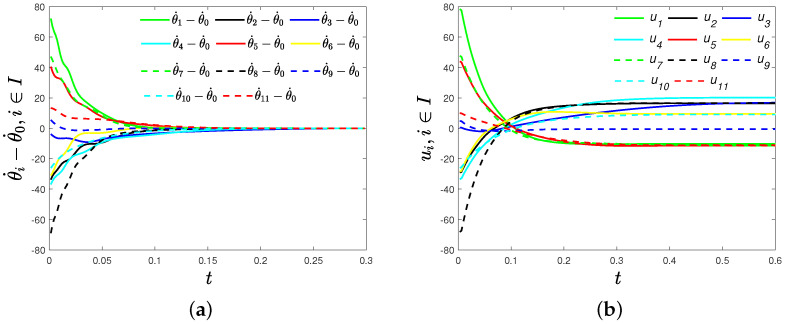
(**a**) Timeevolutions of frequencies differences ${\dot{\theta }}_{i}$-${\dot{\theta }}_{0}$(i∈I) under the distributed control strategy (8); (**b**) time evolutions of the distributed control strategy (8).

Secondly, we verify Theorem 2. Set $\left(\omega_{0},\omega_{1},\omega_{2},\cdots ,\omega_{11}\right)={\left(2,-4,-3.2,-2.4,-1.6,-0.8,0,0.8,1.6,2.4,3.2,4\right)}^{T}$. Choose $\alpha_{1}=0.5$, $\alpha_{2}=1.5$ and $f_{i}=g_{i}=2\;\left(i\in I\right)$. Obviously, $\lambda_{\min}\left(L_{B}-L_{A}\right)=0$. By (9), we can calculate the upper bound of settling time $T_{0}$ = 2.8710 s. In Figure 3a, we can find frequency differences ${\dot{\theta }}_{i}-{\dot{\theta }}_{0}\;\left(i\in I\right)$ that converge to zero, and frequency synchronization is achieved about 0.2840 s < $T_{0}$, which means that fixed-time frequency synchronization is achieved. Time evolutions of the distributed control strategy (8) of each oscillator are shown in Figure 3b.

**Figure 4:**
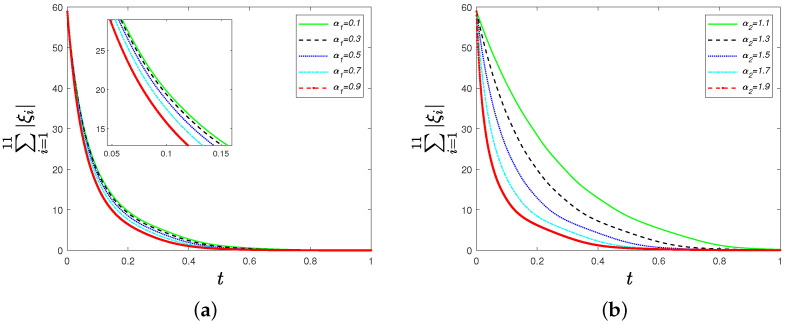
(**a**) Time evolutions of $\sum_{i=1}^{11}\left|\xi_{i}\right|$ under the distributed control strategy (6) for different parameters $\alpha_{1}=0.1,0.3,0.5,0.7,0.9$; (**b**) time evolutions of $\sum_{i=1}^{11}\left|\xi_{i}\right|$ under the distributed control strategy (6) for different parameters $\alpha_{2}=1.1,1.3,1.5,1.7,1.9$.

**Figure 5:**
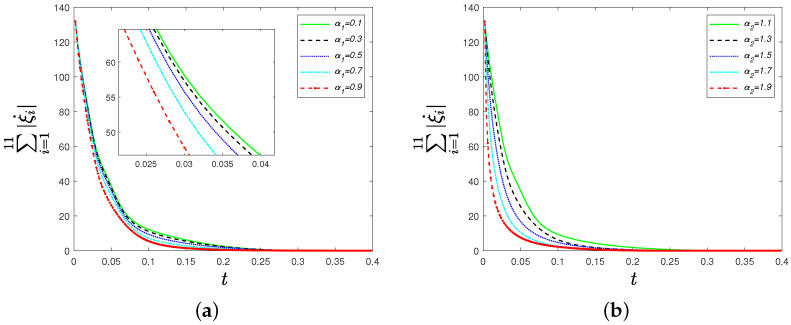
(**a**) Time evolutions of $\sum_{i=1}^{11}\left|{\dot{\xi }}_{i}\right|$ under the distributed control strategy (8) for different parameters $\alpha_{1}=0.1,0.3,0.5,0.7,0.9$; (**b**) time evolutions of $\sum_{i=1}^{11}\left|{\dot{\xi }}_{i}\right|$ under the distributed control strategy (8) for different parameters $\alpha_{2}=1.1,1.3,1.5,1.7,1.9$.

Thirdly, we move to see the influence of parameters $\alpha_{1}$ and $\alpha_{2}$ on $\sum_{i=1}^{11}\left|\xi_{i}\right|$. We choose $\alpha_{1}=0.1,0.3,0.5,0.7,0.9$ and $\alpha_{2}=1.1,1.3,1.5,1.7,1.9$. Figure 4 shows the convergence rate of $\sum_{i=1}^{11}\left|\xi_{i}\right|$ increases as parameter $\alpha_{1}$ or $\alpha_{2}$ grows for identical oscillators. Similarly, the influence of parameters $\alpha_{1}$ and $\alpha_{2}$ on $\sum_{i=1}^{11}\left|{\dot{\xi }}_{i}\right|$ is reflected in Figure 5. We can see that the convergence rate of $\sum_{i=1}^{11}\left|{\dot{\xi }}_{i}\right|$ increases as parameter $\alpha_{1}$ or $\alpha_{2}$ grows for non-identical oscillators.

Finally, we discuss the upper bounds of synchronization time $T_{0}$ for different parameters $\alpha_{1}$ and $\alpha_{2}$. Figure 6 shows that the upper bounds of synchronization time $T_{0}$ increase as $\alpha_{1}$ grows and decrease as $\alpha_{2}$ grows.

## 5. Conclusions

In this paper, the fixed-time phase agreement and frequency synchronization of Kuramoto–oscillator networks with a pacemaker have been investigated. Two distributed control strategies are designed to explore fixed-time synchronization for identical and non-identical oscillators. Furthermore, the upper bounds of synchronization time, which is not related to initial states, have been estimated. Numerical examples have validated the effectiveness of the derived theoretical results. 

## Data Availability

Not applicable.

## Appendix A. Proof of Theorem 1

**Proof.** As $\xi_{i}=\theta_{i}-\theta_{0}$, then ${\dot{\xi }}_{i}={\dot{\theta }}_{i}-{\dot{\theta }}_{0}={\dot{\theta }}_{i}-\omega_{0}$. Thus, network (5) with distributed control strategy (6) becomes

(A1) $${\dot{\xi }}_{i}=\sum_{j=1}^{N}a_{ij}\sin\left(\xi_{j}-\xi_{i}\right)+\sum_{j=1}^{N}b_{ij}\left(\xi_{j}-\xi_{i}\right)-f_{i}sig{\left(\xi_{i}\right)}^{\alpha_{1}}-g_{i}sig{\left(\xi_{i}\right)}^{\alpha_{2}}.$$

Consider the following Lyapunov functional candidate:

$$V_{1}=\frac{1}{2}\xi^{T}\xi =\frac{1}{2}\sum_{i=1}^{N}\xi_{i}^{2}.$$

The derivation of $V_{1}$ along trajectories (A1) gives

(A2) $$\begin{array}{cc} {\dot{V}}_{1} & =\sum_{i=1}^{N}\xi_{i}{\dot{\xi }}_{i}\\ \; & =\sum_{i=1}^{N}\sum_{j=1}^{N}a_{ij}\xi_{i}\sin\left(\xi_{j}-\xi_{i}\right)+\sum_{i=1}^{N}\sum_{j=1}^{N}b_{ij}\xi_{i}\sin\left(\xi_{j}-\xi_{i}\right)-\sum_{i=1}^{N}f_{i}\xi_{i}sig{\left(\xi_{i}\right)}^{\alpha_{1}}\\ \; & \;-\sum_{i=1}^{N}g_{i}\xi_{i}sig{\left(\xi_{i}\right)}^{\alpha_{2}}. \end{array}$$

According to Lemma 1 and the fact $\frac{\sin(\theta_{j}\;-\;\theta_{i})}{\left(\theta_{j}\;-\;\theta_{i}\right)}\in \left[\cos\gamma ,1\right]$, we obtain

(A3) $$\begin{array}{cc} \sum_{i=1}^{N}\sum_{j=1}^{N}a_{ij}\xi_{i}\sin\left(\xi_{j}-\xi_{i}\right) & =\frac{1}{2}\sum_{i=1}^{N}\sum_{j=1}^{N}a_{ij}\left(\xi_{i}-\xi_{j}\right)\sin\left(\xi_{j}-\xi_{i}\right)\\ \; & =-\frac{1}{2}\sum_{i=1}^{N}\sum_{j=1}^{N}a_{ij}\frac{\sin(\xi_{j}-\xi_{i})}{\left(\xi_{j}-\xi_{i}\right)}{\left(\xi_{j}-\xi_{i}\right)}^{2}\\ \; & \leq -\frac{\cos\gamma }{2}\cdot \sum_{i=1}^{N}\sum_{j=1}^{N}a_{ij}{\left(\xi_{j}-\xi_{i}\right)}^{2}\\ \; & =-\cos\gamma \cdot \xi^{T}L_{A}\xi . \end{array}$$

In addition,

(A4) $$\sum_{i=1}^{N}\sum_{j=1}^{N}b_{ij}\xi_{i}\left(\xi_{j}-\xi_{i}\right)=-\frac{1}{2}\sum_{i=1}^{N}\sum_{j=1}^{N}b_{ij}{\left(\xi_{j}-\xi_{i}\right)}^{2}=-\xi^{T}L_{B}\xi ,$$

(A5) $$\sum_{i=1}^{N}f_{i}\xi_{i}\mathrm{sig}{\left(\xi_{i}\right)}^{\alpha_{1}}=\sum_{i=1}^{N}f_{i}\xi_{i}\mathrm{sign}\left(\xi_{i}\right){\left|\xi_{i}\right|}^{\alpha_{1}}\leq \sum_{i=1}^{N}f_{i}{\left|\xi_{i}\right|}^{1+\alpha_{1}},$$

(A6) $$\sum_{i=1}^{N}g_{i}\xi_{i}\mathrm{sig}{\left(\xi_{i}\right)}^{\alpha_{2}}=\sum_{i=1}^{N}g_{i}\xi_{i}\mathrm{sign}\left(\xi_{i}\right){\left|\xi_{i}\right|}^{\alpha_{2}}\leq \sum_{i=1}^{N}g_{i}{\left|\xi_{i}\right|}^{1+\alpha_{2}}.$$

Combining (A3)–(A6), Equation (A2) becomes

$${\dot{V}}_{1}\leq -\xi^{T}\left(\cos\gamma \cdot L_{A}+L_{B}\right)\xi -\sum_{i=1}^{N}f_{i}{\left|\xi_{i}\right|}^{1+\alpha_{1}}-\sum_{i=1}^{N}g_{i}{\left|\xi_{i}\right|}^{1+\alpha_{2}}.$$

If $\lambda_{\min}\left(\cos\gamma \cdot L_{A}+L_{B}\right)\geq 0$, we obtain

$$\begin{array}{cc} {\dot{V}}_{1} & \leq -\sum_{i=1}^{N}f_{i}{\left|\xi_{i}\right|}^{1+\alpha_{1}}-\sum_{i=1}^{N}g_{i}{\left|\xi_{i}\right|}^{1+\alpha_{2}}\\ \; & \leq -f_{\min}2^{\frac{1+\alpha_{1}}{2}}{\left[\frac{1}{2}\sum_{i=1}^{N}{\left|\xi_{i}\right|}^{2}\right]}^{\frac{1+\alpha_{1}}{2}}-g_{\min}2^{\frac{1+\alpha_{2}}{2}}{\left[\frac{1}{2}\sum_{i=1}^{N}{\left|\xi_{i}\right|}^{2}\right]}^{\frac{1+\alpha_{2}}{2}}\\ \; & =-2^{\frac{1+\alpha_{1}}{2}}f_{\min}{V_{1}}^{\frac{1+\alpha_{1}}{2}}-2^{\frac{1+\alpha_{2}}{2}}g_{\min}{V_{1}}^{\frac{1+\alpha_{2}}{2}}. \end{array}$$

By Lemma 2 and Definition 1, the fixed-time phase agreement can be achieved with the settling time bounded by

$$\begin{array}{cc} T_{1} & \leq \frac{1}{2^{\frac{1+\alpha_{1}}{2}}\left(1-\frac{1+\alpha_{1}}{2}\right)f_{\min}}+\frac{1}{2^{\frac{1+\alpha_{2}}{2}}\left(\frac{1+\alpha_{2}}{2}-1\right)g_{\min}}\\ \; & =\frac{1}{2^{\frac{\alpha_{1}-1}{2}}\left(1-\alpha_{1}\right)f_{\min}}+\frac{1}{2^{\frac{\alpha_{2}-1}{2}}\left(\alpha_{2}-1\right)g_{\min}}. \end{array}$$

□

## Appendix B. Proof of Theorem 2

**Proof.** Under the control strategy (8), the derivation of (1) becomes

(A7) $$\begin{array}{cc} {\ddot{\xi }}_{i}={\ddot{\theta }}_{i} & =\sum_{j=1}^{N}a_{ij}\cos\left(\xi_{j}-\xi_{i}\right)\left({\dot{\xi }}_{j}-{\dot{\xi }}_{i}\right)+{\dot{u}}_{i}\\ \; & =\sum_{j=1}^{N}a_{ij}\cos\left(\xi_{j}-\xi_{i}\right)\left({\dot{\xi }}_{j}-{\dot{\xi }}_{i}\right)+\sum_{j=1}^{N}b_{ij}\left({\dot{\xi }}_{j}-{\dot{\xi }}_{i}\right)-f_{i}sig{\left({\dot{\xi }}_{i}\right)}^{\alpha_{1}}-g_{i}sig{\left({\dot{\xi }}_{i}\right)}^{\alpha_{2}}. \end{array}$$

Consider the following Lyapunov functional candidate:

$$V_{2}=\frac{1}{2}\dot{\xi^{T}}\dot{\xi }=\frac{1}{2}\sum_{i=1}^{N}{\dot{\xi_{i}}}^{2}.$$

The derivation of $V_{2}$ along trajectories (A7) gives

(A8) $$\begin{array}{cc} {\dot{V}}_{2} & =\sum_{i=1}^{N}\dot{\xi_{i}}{\ddot{\xi }}_{i}\\ \; & =\sum_{i=1}^{N}{\dot{\xi }}_{i}\left[\sum_{j=1}^{N}a_{ij}\cos\left(\xi_{j}-\xi_{i}\right)\left({\dot{\xi }}_{j}-{\dot{\xi }}_{i}\right)+\sum_{j=1}^{N}b_{ij}\left({\dot{\xi }}_{j}-{\dot{\xi }}_{i}\right)-f_{i}sig{\left({\dot{\xi }}_{i}\right)}^{\alpha_{1}}-g_{i}sig{\left({\dot{\xi }}_{i}\right)}^{\alpha_{2}}\right]\\ \; & =\sum_{i=1}^{N}\sum_{j=1}^{N}{\dot{\xi }}_{i}a_{ij}\cos\left(\xi_{j}-\xi_{i}\right)\left({\dot{\xi }}_{j}-{\dot{\xi }}_{i}\right)+\sum_{i=1}^{N}\sum_{j=1}^{N}b_{ij}{\dot{\xi }}_{i}\left({\dot{\xi }}_{j}-{\dot{\xi }}_{i}\right)-\sum_{i=1}^{N}f_{i}{\dot{\xi }}_{i}sig{\left({\dot{\xi }}_{i}\right)}^{\alpha_{1}}\\ \; & \;-\sum_{i=1}^{N}g_{i}{\dot{\xi }}_{i}sig{\left({\dot{\xi }}_{i}\right)}^{\alpha_{2}}. \end{array}$$

According to Lemma 1 and the fact $\left|\cos(\theta_{j}-\theta_{i})\right|\leq 1$, we can obtain

(A9) $$\begin{array}{cc} \sum_{i=1}^{N}\sum_{j=1}^{N}{\dot{\xi }}_{i}a_{ij}\cos\left(\xi_{j}-\xi_{i}\right)\left({\dot{\xi }}_{j}-{\dot{\xi }}_{i}\right) & =-\frac{1}{2}\sum_{i=1}^{N}\sum_{j=1}^{N}a_{ij}{\left({\dot{\xi }}_{j}-{\dot{\xi }}_{i}\right)}^{2}\cos\left(\xi_{j}-\xi_{i}\right)\\ \; & \leq \frac{1}{2}\sum_{i=1}^{N}\sum_{j=1}^{N}a_{ij}{\left({\dot{\xi }}_{j}-{\dot{\xi }}_{i}\right)}^{2}\\ \; & ={\dot{\xi }}^{T}L_{A}\dot{\xi }. \end{array}$$

In addition,

(A10) $$\begin{array}{c} \sum_{i=1}^{N}\sum_{j=1}^{N}b_{ij}{\dot{\xi }}_{i}\left({\dot{\xi }}_{j}-{\dot{\xi }}_{i}\right)=-\frac{1}{2}\sum_{i=1}^{N}\sum_{j=1}^{N}b_{ij}{\left({\dot{\xi }}_{j}-{\dot{\xi }}_{i}\right)}^{2}=-{\dot{\xi }}^{T}L_{B}\dot{\xi }, \end{array}$$

(A11) $$\begin{array}{c} \sum_{i=1}^{N}f_{i}{\dot{\xi }}_{i}\mathrm{sig}{\left({\dot{\xi }}_{i}\right)}^{\alpha_{1}}=\sum_{i=1}^{N}f_{i}{\dot{\xi }}_{i}sign\left({\dot{\xi }}_{i}\right){\left|{\dot{\xi }}_{i}\right|}^{\alpha_{1}}\leq \sum_{i=1}^{N}f_{i}{\left|{\dot{\xi }}_{i}\right|}^{1+\alpha_{1}}, \end{array}$$

(A12) $$\begin{array}{c} \sum_{i=1}^{N}g_{i}{\dot{\xi }}_{i}sig{\left({\dot{\xi }}_{i}\right)}^{\alpha_{2}}=\sum_{i=1}^{N}g_{i}{\dot{\xi }}_{i}sign\left({\dot{\xi }}_{i}\right){\left|{\dot{\xi }}_{i}\right|}^{\alpha_{2}}\leq \sum_{i=1}^{N}g_{i}{\left|{\dot{\xi }}_{i}\right|}^{1+\alpha_{2}}. \end{array}$$

Combining (A9)–(A12), Equation (A8) becomes

$${\dot{V}}_{2}\leq -{\dot{\xi }}^{T}\left(L_{B}-L_{A}\right)\dot{\xi }-\sum_{i=1}^{N}f_{i}{\left|{\dot{\xi }}_{i}\right|}^{1+\alpha_{1}}-\sum_{i=1}^{N}g_{i}{\left|{\dot{\xi }}_{i}\right|}^{1+\alpha_{2}}.$$

If $\lambda_{\min}\left(L_{B}-L_{A}\right)\geq 0$, we obtain

$$\begin{array}{cc} {\dot{V}}_{2} & \leq -\sum_{i=1}^{N}f_{i}{\left|{\dot{\xi }}_{i}\right|}^{1+\alpha_{1}}-\sum_{i=1}^{N}g_{i}{\left|{\dot{\xi }}_{i}\right|}^{1+\alpha_{2}}\\ \; & =-\sum_{i=1}^{N}f_{i}{\left[{\left|{\dot{\xi }}_{i}\right|}^{2}\right]}^{\frac{1+\alpha_{1}}{2}}-\sum_{i=1}^{N}g_{i}{\left[{\left|{\dot{\xi }}_{i}\right|}^{2}\right]}^{\frac{1+\alpha_{2}}{2}}\\ \; & =-f_{\min}2^{\frac{1+\alpha_{1}}{2}}{\left[\frac{1}{2}\sum_{i=1}^{N}{\left|{\dot{\xi }}_{i}\right|}^{2}\right]}^{\frac{1+\alpha_{1}}{2}}-g_{\min}2^{\frac{1+\alpha_{2}}{2}}{\left[\frac{1}{2}\sum_{i=1}^{N}{\left|{\dot{\xi }}_{i}\right|}^{2}\right]}^{\frac{1+\alpha_{2}}{2}}\\ \; & =-2^{\frac{1+\alpha_{1}}{2}}f_{\min}V_{2}^{{}^{\frac{1+\alpha_{1}}{2}}}-2^{\frac{1+\alpha_{2}}{2}}g_{\min}V_{2}^{{}^{\frac{1+\alpha_{2}}{2}}}. \end{array}$$

By Lemma 2 and Definition 2, network (1) with non-identical oscillators under the distributed control strategy (8) achieves fixed-time frequency synchronization with the settling time bounded by

$$\begin{array}{cc} T_{2} & \leq \frac{1}{2^{\frac{1+\alpha_{1}}{2}}\left(1-\frac{1+\alpha_{1}}{2}\right)f_{\min}}+\frac{1}{2^{\frac{1+\alpha_{2}}{2}}\left(\frac{1+\alpha_{2}}{2}-1\right)g_{\min}}\\ \; & =\frac{1}{2^{\frac{\alpha_{1}-1}{2}}\left(1-\alpha_{1}\right)f_{\min}}+\frac{1}{2^{\frac{\alpha_{2}-1}{2}}\left(\alpha_{2}-1\right)g_{\min}}. \end{array}$$

□
